# Correspondence

**Published:** 1888-07

**Authors:** E. P. Beadles

**Affiliations:** Danville, Va.


### CORRESPONDENCE.

DANVILLE, VA., July 4, 1888.

EDITOR DENTAL REGISTER:

An "agent" for a man who has invented a wonderful "local anæsthetic" for the painless extraction of teeth has been traveling through this State selling rights to dentists for the use of this medicine. This agent has been to my office, and we operated on a number of cases, but I am too old a hand with cocaine not to recognize this drug as the anæsthetic part of his "mixture." He kindly offered to sell me the "right" to use this medicine in my town for seventy-five dollars. Several dentists of my acquaintance have bought his formula, but I am very sure that I can furnish one free which will do all that this new medicine will do. All the symptoms, after each injection, were manifestly those of cocaine, the numbness of the tongue, the bitter taste, and in some cases the buzzing in the head. In fact several of the patients operated upon readily recognized cocaine as it had been used upon them before. I am quite satisfied from my experience with each medicine, that the inventor of this new anæsthetic has gotten up a simple formula (the odor of carbolic acid is plain) in which the now old drug cocaine is the principal part, and is humbugging the dentists through the country into buying it. I state these facts, and my own impressions, in order that those who may be approached may not act blindly, as one who has never experimented with cocaine can easily be deceived and made to think that he has found a good thing, as cocaine is in very many cases.

E. P. BEADLES.

